# Multi-Systemic Biological Risk and Cancer Mortality: The NHANES III Study

**DOI:** 10.1038/s41598-020-61945-9

**Published:** 2020-03-19

**Authors:** Teofilia Acheampong, Luohua Jiang, Argyrios Ziogas, Andrew O. Odegaard

**Affiliations:** 1 Department of Epidemiology, Mailman School of Public Health, Columbia University, 722 W 168th street, New York, NY 10032 United States; 2 Department of Epidemiology, University of California-Irvine, Anteater Instruction & Research Building (AIRB), 653 E. Peltason Drive, Suite 3060 E, Irvine, CA 92697 United States

**Keywords:** Cancer epidemiology, Human behaviour, Predictive markers

## Abstract

Multi-systemic biological risk (MSBR), a proxy for allostatic load, is a composite index of biomarkers representing dysregulation due to responses to chronic stress. This study examined the association of an MSBR index with cancer mortality. The sample included n = 13,628 adults aged 20–90 from the NHANES III Linked Mortality File (1988–1994). The MSBR index included autonomic (pulse rate, blood pressure), metabolic (HOMA_ir_, triglycerides, waist circumference), and immune (white blood cell count, C-reactive protein) markers. We fit Cox proportional hazards models to estimate hazard ratios (HRs) and 95% confidence intervals (CI) of overall cancer mortality risk, according to quartiles (q) of the index. In multivariable models, compared to those in q1, q4 had a 64% increased risk for cancer mortality (HR = 1.64, 95% CI:1.13–2.40). The immune domain drove the association (HR per unit = 1.19, 95% CI:1.07–1.32). In stratified analyses, the HR for those with a BMI ≥ 25 was 1.12 per unit (95% CI:1.05–1.19) and those with a BMI < 25 was 1.04 per unit (95% CI:0.92–1.18). MSBR is positively associated with risk for cancer mortality in a US sample, particularly among those who are overweight or obese. The utilization of standard clinical measures comprising this index may inform population cancer prevention strategies.

## Introduction

Multi-systemic biological risk (MSBR) is a proxy for allostatic load (AL). It is a metric of health risk that captures the complex biological cascade that occurs in autonomic, metabolic, and immune domains in response to chronic environmental and psychosocial stress^1–3^. The validity of the AL construct is established and demonstrates common variance and statistical coherence between, prominent primary mediators of the stress response (i.e., stress hormones), and secondary mediators reflecting the resulting biological alterations in autonomic, metabolic, and immune domains that accumulate over time^4–8^. Importantly, summary AL indices have demonstrated a non-additive, stronger magnitude of association with outcomes compared with the individual components of the index^9^.

Previous research has shown that autonomic, metabolic, and immune disorders, share common risk factors with cancer outcomes^10–12^. Moreover, there is a strong physiological link between over-activation of the stress response and regulation of the tumor microenvironment^13–15^. In mechanistic animal studies where stress can demonstrably be reproduced, chronic responses to stress influence processes involved in tumorigenesis^16–19^. However, observational studies assessing self-report of stressful life events and cancer outcomes have generally been inconsistent^20–25^.

Epidemiological studies show positive associations between higher levels of AL indices and cardiovascular disease (CVD), as well as mortality risk^26,27^. However, we are not aware of any studies that have examined an index of AL with cancer outcomes. With cancer being the second leading cause of death in the U.S. and globally, having shifting underlying contributors to this burden^28–30^, a prospective analysis examining the association of an index of MSBR with cancer mortality would address a major gap in the literature. It would also allow for the ability to triangulate population-level evidence with *in vivo* and *in vitro* studies of stress and cancer outcomes^14,31,32^. Furthermore, it may also have clinical utility for cancer prediction, as the index relies upon commonly measured biomarkers to address this gap. In this study, we prospectively examined the association between an index of MSBR and cancer mortality utilizing the mortality linked NHANES III study, a representative sample of the U.S. from 1988 to 1994. We also examined the relative contribution of each sub-domain, in the relationship between MSBR and cancer death.

## Results

The total person-years of follow-up among 13,628 study participants were 269, 074.7.6 years (mean follow-up of 21 years), and 7.2% (n = 978) of the study population died due to malignant neoplasms during the follow-up period. The weighted mean age at baseline was 44.4 (SD, 16.8) years. Table 1 displays participant characteristics across quartiles of the MSBR index. About 12% of the study population fasted less than the requested 6 hours before venous blood collection. Compared to quartile 1, participants from quartile 2–4 were older, with a higher proportion being female, less educated, less likely to have health insurance, less physically active, and had higher average BMIs.

**Table 1:** Baseline Characteristics of Participants According to Quartiles of Multi-Systemic Biological Risk (n = 13,628), NHANES III, 1994–1998.

	Quartile 1	Quartile 2	Quartile 3	Quartile 4
Score Range	(0–4)	(5–6)	(7–8)	(9–14)
	(n = 4,745)	(n = 3,827)	(n = 2,908)	(n = 2,148)
Median Age (IQR 25–75)	35 (27–47)	45 (32–65)	53 (37–67)	58 (43–69)
Sex (% Female)	46.6	51.5	53.5	59.7
% Non-Latino White	78.0	75.5	74.4	75.4
% Non-Latino Black	9.8	10.6	10.6	11.4
% Mexican American	4.3	5.1	6.2	6.2
% Other	7.8	8.7	8.6	6.9
% High school or less	48.0	60.0	67.5	74.4
% Without health insurance	19.1	22.2	25.2	27.0
% Within urban area	51.8	50.0	47.2	40.7
^b^% Little to no physical activity	24.4	28.1	36.9	45.3
^c^% Currently smoking	31.9	35.3	32.4	28.8
^d^% Using ≥1 medication	3.2	12.7	23.9	50.6
^a^Diet score (HEI)	63.7 (0.36)	63.2 (0.35)	63.5 (0.54)	64.5 (0.55)
^a^Alcohol, drinks/week	4.3 (0.20)	4.1 (0.27)	3.1 (0.31)	2.4 (0.30)
^a^BMI	23.4 (0.07)	26.6 (0.11)	29.8 (0.11)	32.6 (0.24)

^a^Mean (Standard Error).

^b^Physically active: <9 METS/week = Little to no physical activity.

^c^Current smoking versus previous history or no smoking.

^d^Use of at least one medication (including high blood pressure, diabetes, cholesterol).

Table 2 presents HRs for cancer mortality by quartiles of the MSBR index. There was a graded, positive association between higher index scores and risk for cancer mortality after adjustment for all covariates (model 4). To inform the interpretation of the main index results, we also assessed the association between the individual domains (immune, metabolic, and autonomic) and risk for cancer mortality. Shown in Table 3, we observed a positive association between the immune domain [HR per higher unit score = 1.19, 95% CI: (1.07–1.32)] after adjustment (model 4). However, there was no association found for the metabolic domain [HR per higher unit score = 1.01, 95% CI: (0.95–1.08)]. Furthermore, the autonomic domain was positive, although the confidence interval included the null [HR per higher unit score = 1.15, 95% CI: (0.98–1.34)].

**Table 2:** Hazard Ratio and 95% CI of Cancer Mortality According to Quartiles of Multi-Systemic Biological Risk (n = 13,628), NHANES III, 1994–1998.

MSBR Groups	HR (95%CI) Model 1: Demographics	HR (95%CI) Model 2: (+SES)	HR (95%CI): Model 3: (+Lifestyle)	HR (95%CI): Model 4: (+BMI)
Quartile 1	Ref	Ref	Ref	Ref
Quartile 2	1.18 (0.90–1.54)	1.15 (0.88–1.50)	1.10 (0.85–1.43)	1.11 (0.85–1.46)
Quartile 3	1.34 (1.03–1.75)	1.28 (0.99–1.66)	1.27 (0.97–1.66)	1.29 (0.92–1.79)
Quartile 4	1.72 (1.22–2.42)	1.61 (1.15–2.25)	1.61 (1.16–2.24)	1.64 (1.13–2.40)
Continuous HR	1.08 (1.03–1.14)	1.07 (1.02–1.12)	1.07 (1.02–1.12)	1.07 (1.01–1.14)
P. Trend	0.001	0.005	0.007	0.02

Model Covariates: (1) fasting status (<6 hours/> = 6 hours), age (continuous), sex (male/female), ethnicity (Non-Latino White/Non-Latino Black/Mexican American), (2) Model 1 + education (High school or less), health insurance coverage (Yes/No), urbanization (% urban), (3) Model 2 + HEI scores (continuous), physical inactivity (% active yes/no), smoking status (current/former/never), alcoholic drinks per week (continuous), medication (any/none), and (4) Model 3 + BMI.

**Table 3:** Hazard Ratio and 95% CI of Cancer Mortality Risk for Domain-Specific Variables of Multi-Systemic Biological Risk (Per unit increase in index) (n = 13,628), NHANES III, 1994–1998.

Domain Variables* of MSBR Index	HR (95%CI) Model 1: Demographics	HR (95%CI): Model 2: (+SES)	HR (95%CI): Model 3: (+Lifestyle)	HR (95%CI): Model 4: (+BMI)
Immune Index Variable	1.30 (1.17–1.45)	1.28 (1.16–1.43)	1.20 (1.07–1.34)	1.19 (1.07–1.32)
Metabolic Index Variable	1.00 (0.96–1.06)	1.00 (0.95–1.05)	1.02 (0.97–1.07)	1.01 (0.95–1.08)
Autonomic Index Variable	1.19 (1.02–1.37)	1.17 (1.01–1.36)	1.15 (0.98–1.34)	1.15 (0.98–1.34)

Model Covariates: (1) fasting status(<6 hours/> = 6 hours), age (continuous), sex (male/female), ethnicity(Non-Latino White/Non-Latino Black/Mexican American), (2) Model 1 + education (High school or less), health insurance coverage (yes/no), urbanization (% urban), (3) Model 2 + HEI scores (continuous), physical inactivity (% active yes/no), smoking status (current/former/never), alcoholic drinks per week(continuous), medication (any/none), and (4) Model 3 + BMI.

*This table includes continuous hazard ratios for each domain and all models include all three of the domain specific index variables.

Analyses used to determine if there was effect measure modification by race, sex, and age displayed no evidence of interaction for those covariates. However, there was evidence that the association differed by BMI in tests for statistical interaction (P = 0.02); therefore, we fitted the models stratified by overweight status (BMI ≥ 25). Among overweight participants (BMI ≥ 25), model 4 displayed positive associations between a higher index score and risk for cancer death (Supplementary Table 2). Furthermore, amongst overweight participants (BMI ≥ 25), in the model assessing domain-specific variables, the immune domain [HR per higher unit score = 1.26, 95% CI: (1.08–1.48)] and the autonomic domain [HR per higher unit score = 1.23, 95% CI: (1.00–1.52)], displayed an increased risk for cancer death (Supplementary Table 3). There was no association found amongst those with BMI < 25, although there was less precision due to fewer cases (Supplementary Table 4).

## Discussion

This study provides evidence for a strong association between an index of MSBR, (a proxy for AL), and cancer mortality in the NHANES population. Additionally, we observed that the immune domain mainly drove the association. Sensitivity analyses also suggested the association was stronger in participants who were overweight or obese.

This study makes a novel contribution as previous studies have reported positive associations between higher MSBR indices and risk for heart disease, as well as overall mortality^27,33,34^. However, there is little evidence of cancer as an outcome. A study by Borell *et al*.^35^ found that after adjustment for sociodemographic variables, mortality rates were 88% higher for participants with the highest AL score. Furthermore, an analysis of a Scottish population reported a higher AL was not associated with any of the specific causes of death over the follow-up period, including cancer death^36^.

A previous study by Gathirua-Mwangi *et al*. assessed whether metabolic syndrome, as well as the individual markers, were associated with total cancer mortality in the NHANES III Study. The presence of metabolic syndrome (yes/no) was associated with a 33% increase in total cancer mortality^37^. In terms of the individual components, the study displayed that only systolic BP and serum glucose were associated with an increased risk of death from total cancer^37^. The current study utilized an updated cancer mortality file and assessed a combination of markers for each biological domain versus a single marker. Furthermore, the AL theory includes an inflammatory aspect, which is a crucial risk factor for cancer outcomes.

The relationship between individual cardiometabolic risk factors and cancer has been reviewed and tested^10,11^. Some cardio-metabolic risk factors such as hypertension, abdominal obesity, insulin resistance, or inflammation have shown positive associations ranging from weak to strong concerning cancer mortality depending on the marker, the cancer type, and the appropriate adjustments for confounding variables^12,38–40^. Moreover, many of the referenced studies present inconsistent and null findings depending on cancer type, gender, or study population.

The idea that the AL index solely reflects metabolic syndrome was previously refuted^9^. Metabolic syndrome is defined as the presence of at least three of five components^41^: high BP, abdominal obesity, elevated fasting glucose, HDL cholesterol, and triglycerides. The MSBR index is defined as the additive combination of the number of physiological measures with values above a high-risk threshold generally used in clinical practice^42,43^: pulse rate, BP, insulin resistance, triglycerides, waist circumference, CRP, and WBC. (Supplementary Table 1). This index incorporates a spectrum of risk, where an increase in the index represents an elevated level of a measure, indicating increased risk^44^.

In terms of mechanism, research in this area describes that prolonged exposure to stress-related hormones may influence various processes involved in tumorigenesis^14,45^, including impaired DNA repair^15,19,46,47^. Furthermore, stress may be permissive by way of immunomodulation or conducive by aiding in alterations of the tumor microenvironment^13,48–50^; this may be through pro- and anti-inflammatory cytokines that are regulated by glucocorticoids and catecholamines^15,51^.

Some limitations warrant further consideration. First, we utilized a one -point in time measurement of the cumulative biological risk index; repeated measures of biomarkers would account for time-varying changes of the index and would reduce the potential for exposure misclassification. Next, the biological markers utilized to create the MSBR index in this study were restricted to availability in NHANES. Also, our analytical sample is a subset of the original cohort due to missing data. Even though we imputed relevant covariates to mitigate bias, participants were excluded if they had missing data on follow up time, fasting time, or any biomarkers included in the AL index. Furthermore, only aggregate information on leading causes of death is available in the public 2015 mortality follow-up, as a result, cancer-specific analyses were not possible. Also, there are well-recognized limitations to the use of a 24-h dietary recall to calculate an HEI score, thus we cannot rule out misclassification of participants concerning HEI status. Physical activity was assessed as an activity done in the last month. While this is maybe a useful marker for pattern of behavior, it is not likely a true representation of a physically fit individual.

For this analysis, we adjusted for an array of potential confounders, yet, we cannot exclude the potential for residual confounding that might result in a less precise or underestimation of the association, particularly factors that were either not measured or measured inadequately. To improve inference, we assessed the robustness of the association to unmeasured confounding by calculating an E-value and corresponding lower 95% CI^52,53^. The E-value for the adjusted association of those in q4 of the MSBR index and cancer mortality was 2.66 and 1.51 for the CI. For those in q4, an unmeasured confounder would need to be associated with both the MSBR index and cancer mortality by a hazard ratio of 2.66 each to explain away the observed positive association, and by 1.51 fold each to shift the lower CI for the estimate to include the null value, beyond the measured covariates. The E-value for the adjusted association between the immune domain and cancer mortality was 1.67 and 1.34 for the CI. This estimate suggests that the association was reasonably robust to unmeasured confounding, and further implies that residual confounding completely explaining the association is unlikely.

In the practical sense, AL is operationalized as a metric of health risk used to express shared physiologic variance in multiple biological systems due to external stressors^54,55^. Our results suggest that, by way of immune and autonomic risk factors, the MSBR index is strongly associated with cancer mortality risk, specifically amongst overweight and obese individuals. The use of the MSBR index, a proxy of AL, may potentially be useful for understanding new directions regarding relevant strategies in cancer prevention. In particular, relevant pathways of the early stages by which external factors may influence physiological functioning and harbor biological environments conducive to the development of aggressive cancer outcomes^8,56–58^. It may also have clinical utility, as the index relies upon commonly measured pre-clinical biomarkers and may be a useful, practical screening tool for high-risk individuals, highlighting early points to intervene and potentially prevent premature cancer death, particularly amongst overweight and obese individuals. Future attempts should be made to replicate and validate similar indices in other populations.

## Methods

### Study population

The NHANES III is a complex, multistage clustered probability sample conducted by the National Center for Health Statistics (NCHS); it includes (n = 33,994) participants who represent the US non-institutionalized population from 1988 to 1994. Detailed descriptions of all NHANES III data collection, analytical guidelines, and full datasets are publicly available at (https://www.cdc.gov/nchs/nhanes/)^59^. In brief, demographic characteristics, medical, family history, dietary and lifestyle factors were collected at study entry from participants through a structured household interview. Physical examinations, including anthropometric measurements and blood samples, were collected within mobile examination centers^59^.

For this study, adults 20 years or older with eligible mortality linkage were included (n = 18,805). Participants were excluded if they reported current pregnancy (n = 231), a history of cancer (n = 780) at baseline, and only completed a modified home examination (n = 455). Participants who died from cancer within two years of the study end date (n = 153), or had missing data on fasting time (n = 1,810) or at least one of the biomarkers included in the AL index (n = 1,788) were also excluded, leaving a final analytic sample of (n = 13,628) participants. Of note, about 12% of the observations in this final analytic sample had missing data for five covariates (insurance, BMI, healthy eating index, alcohol, and education), in which case we used multiple imputation. All participants within NHANES III provided written informed consent, and the NHANES study was approved by the National Center for Health Statistics (NCHS) Institutional Review Board. For this analysis, we used publicly available data without personal identifiable information and all methods were carried out in accordance with relevant guidelines and regulations.

### Case ascertainment

Mortality status for the survey participants was ascertained primarily through the NHANES III mortality file linked with the National Death Index (NDI)^60^. Person-months of follow-up were calculated from the baseline interview date through the registered date of death or end of study period on the 31^st^ of December 2015. Underlying causes of death were identified from the Underlying Leading Cause of Death Recode using International Classification of Diseases (ICD) from both the Ninth Revision (ICD-9) and the Tenth Revision (ICD-10 for deaths after 1998) coding that span across the years. The primary outcome of this study was all cancer‐specific death, as defined by UCOD_LEADING codes (C00-C97)^60^. The corresponding ICD-9 and ICD-10 coded can be found in a previous report^61^. Only aggregate information on leading causes of death is available in the public 2015 mortality follow-up, therefore cancer-specific analyses are not possible^60^.

### Exposure assessment

The MSBR index is based on an aggregate score of seven biomarkers across multiple regulatory systems theorized to represent the overall extent of physiological dysregulation or AL. Our operationalization of the index was similar to other studies that have investigated MSBR and AL using a clinically or empirically significant threshold^3,43,62,63^. The domains and biomarkers for the index include: autonomic (pulse rate, blood pressure (BP)), metabolic (Homeostasis model assessment (HOMA-_IR_), triglycerides, and waist circumference), and immune (white blood cell count (WBC), C-reactive protein (CRP)) domains. Participants were assigned a score for each biomarker informed by either clinical cut points or based upon evidence in the literature indicating a threshold of risk for disease (Supplementary Table 1). Each biomarker within each domain was assigned a value of either a 0 (no or decreased risk), 1(moderate), or 2 (high). Waist circumference was scored based on sex-specific thresholds. The value for each marker was then summed by subdomain, and then further aggregated to a final index for each participant. We then ranked the total index (range 0–14) into quartiles, and a higher score represents a higher presence of dysregulation.

Participants were asked to fast at least 6 hours for venous blood collections, and the amount of hours fasted was ascertained from each participant before lab draws^64^. Details regarding specimen collection and laboratory procedures are documented in the NHANES III Laboratory Procedures manual^64^. Homeostasis model assessment (HOMA-_IR_) was used to estimate insulin resistance according to the formula: fasting serum insulin level (μU/mL) × fasting plasma glucose level (mmol/L)/22.5, where the higher the HOMA-_IR_ value, the more insulin resistant the individual^65^.

### Covariates

We obtained additional information on characteristics a priori that would be associated with stress or biomarker level and cancer mortality based on previous research. Race/ethnicity was categorized as non-Hispanic white, non-Hispanic black, and Mexican American or “other” for participants who did not identify with one of these categories. Other covariates included age, sex, current tobacco use (cotinine level >10 ng/mL^66^ or self-report current smoker), years of education (≤12 years/greater than 12 years), health insurance (yes/no), alcoholic drinks per week and fasting status (fasted >= 6 hours before venous blood collection/<6 hours). Body mass index (BMI) was calculated by the formula weight (kg)/height (m^2^), and further categorized as underweight for a BMI under 18.5, normal or healthy weight as 18.5–24.9, overweight as 25.0–29.9, and obese as 30.0 and above^67^. Geographic urbanization classification was based on USDA Rural/Urban continuum codes, where urban includes metro areas with a population of ≥1 million^68^. Within NHANES III, participants were asked how many times in the previous month did they engage in specified physical activities^69^. Each participants physical activity was assigned an intensity value by NHANES (metabolic equivalent tasks [METs]), representing the ratio of the energy expenditure of the activity to the basal metabolic rate^70^. We, therefore, measured physical activity by: (Number of times engaged in specific physical activity in previous month × MET assignment)/4 weeks). Participants were classified as physically active for >15 METS per week, moderately active for 9–15 METS per week, and little to no physical activity for <than 9 METS per week^70,71^. Diet as a confounder was estimated by calculating the Healthy Eating Index (HEI) from the NHANES III dietary intake data, which provides a measure of the overall quality of an individual’s diet by alignment with Dietary Guidelines^72^. A score represents the sum of ten diet components (grain, fruit, vegetables, dairy, and meat food groups; intake of dietary fats, saturated fats, cholesterol, and sodium; and a variety score). For the first five components, a score of 0 was assigned when no foods in a particular group were eaten. For intake of dietary fats, saturated fats, cholesterol, and sodium, participants with an intake at the recommended level received a maximum score of 10^72^. Each component has a scoring range of 0 to 10, and total scores ranged from 0–100. Medication for type 2 diabetes, high BP, and high cholesterol were also assessed.

### Statistical analysis

NHANES III utilizes a complex survey design. To take this into account, we utilized the appropriate variables for the design effects of stratification and clustering. Furthermore, estimates were weighted to adjust for the differential probabilities of sampling and non-response, to represent the total civilian, non-institutionalized US population as per NHANES documentation. Stata utilizes Taylor Series Linearization for calculating standard errors and 95%CI for means and percentages. Study characteristics were described by the MSBR index, using means and standard errors for continuous variables and percentages for categorical variables. Cox proportional hazard regressions were used to estimate HRs and 95% CI for associations between quartiles of the MSBR index and overall cancer mortality. We assessed tests for trend by including the index modeled as a continuous variable in the Cox models. We also evaluated the association between each domain and cancer mortality while mutually adjusting for each domain. Using both imputed and non-imputed data, we assessed whether each covariate met the proportional hazards assumption by modeling a term for interaction between the natural log of time and each covariate. Tests of the proportional hazards assumption did not indicate any departures from proportional hazards (P > 0.10 for all).

We built four models to provide statistical inference. Model 1 included demographic variables (age, sex, race/ethnicity) and fasting status. Model 2 included variables from model 1 plus socioeconomic variables (education, health insurance, geographic urbanization). Model 3 includes variables from model 2 plus lifestyle variables (physical activity, current tobacco use, the HEI, alcoholic drinks per week, and medication use). Finally, model 4 adjusted for all variables previously mentioned and BMI categories. Secondarily, we tested for effect measure modification by age group (20–35, 36–50, 50–65, 65+), sex (male vs. female), race-ethnicity (non-Latino White, Non-Latino Black, Mexican American, Other), and BMI categories (<18.5, >=18.5–24.9, 25–29.9, >30 and <25 vs. >=25), by including a cross-product term with each covariate and the continuous MSBR index in separate models and stratified results were presented if there was evidence of differences. Models were tested for multicollinearity by computing the variance inflation factor for all variables. We also calculated an E-value for quartile 4 of the MSBR index as well as the immune domain adjusted estimates (model 4) and corresponding lower 95% CI to assess the robustness of the association to unmeasured confounding^52,53^.

About 88% of the observations had complete data for all the variables relevant to this study. We utilized multiple imputation using chained equations (MICE), to generate 20 imputed datasets for the estimation of 5 covariates: healthy eating index (missing = 3%), education (missing = 0.5%), health insurance (missing = 5%), alcoholic drinks per week (missing = 3.4%), and BMI (missing = 0.05%) using all other relevant, complete variables within the analysis^73–75^. To obtain Cox regression estimates from the multiply imputed data, we used Rubin’s combination rules as tested and suggested by NHANES documentation. We conducted a sensitivity analysis utilizing the participants with complete data only, and the results did not remarkably differ from the imputed analysis, other than improved precision. Therefore, the entire analysis was carried out with imputed estimates. We utilized both statistical software, SAS 9.4, and STATA 14.

## Supplementary information

Supplementary Tables.
