# Supplementary material for: Multi-Systemic Biological Risk and Cancer Mortality: The NHANES III Study
Source: Sci Rep. 2020 Mar 19;10:5047. doi: 10.1038/s41598-020-61945-9 (PMC7081240; doi:10.1038/s41598-020-61945-9)
Supplement: Supplementary file 1 — Supplementary Tables. [file 41598_2020_61945_MOESM1_ESM.docx]

**Supplementary Material**

Multi-Systemic Biological Risk and Cancer Mortality: The NHANES III Study

Authors: Teofilia Acheampong^1^, Luohua Jiang^2^, Argyrios Ziogas^2^, Andrew O. Odegaard^2†^.

1. Department of Epidemiology, Mailman School of Public Health, Columbia University, New York, NY, USA

2. Department of Epidemiology, University of California-Irvine, Anteater Instruction & Research Building (AIRB). 653 E. Peltason Dr. (3^rd^ Floor) Suite 3060 E

Irvine, CA 92697, USA

*[aodegaar@uci.edu](mailto:aodegaar@uci.edu)

| **Supplementary Table 1. Score assignment for each biological measurement** | | | | |
| --- | --- | --- | --- | --- |
| Measurement | Score = 0 | Score=1 | Score = 2 | Supplemental References |
| Pulse Rate (Beats/Min) | ≤60 | 61-99 | ≥ 100 | ^1,2^ |
| Blood Pressure (mm/Hg) | <120/80 | 120-139/80-89 | ≥140/90mm/Hg or hypertension medication | ^2–4^ |
| Homa-_IR_ | <2.6 | ≥2.6-4.65 | >4.65 or diabetes diagnosis | ^5–7^ |
| Triglycerides | <150 mg/dL | 150 to 199 mg/dL | > 200 mg/dL | ^8^ |
| Waist Circumference | < 94 cm (M), or < 80 cm (W), | 94 - 102 cm (M) or 80-88 cm (W) | >102 cm (M) or >88 cm (W) | ^9^ |
| White Blood Cell Count | 1500-4500 cells/mcL | 4500-11,000 cell/mcL | >11,001 cells/mcL or <1500 | ^10–12^ |
| C-Reactive Protein | ≤.21mg/dL | >.21-1mg/dL | >1 mg/dL | ^13^ |
| M=Men  W=Women | | | | |

| **Supplementary Table 2. Hazard Ratio and 95% CI of Cancer Mortality Risk According to Quartiles of Multi-Systemic Biological Risk stratified by BMI (< 25 vs. ≥ 25 kg/m^2^) NHANES III, 1994-1998** | | | | |
| --- | --- | --- | --- | --- |
| MSBR Groups | (Cancer deaths/ Total) | HR (95%CI)  Model 1: (+Demographics) | HR (95%CI)  Model 2:  (+ SES) | HR (95%CI)  Model 3:  (+Lifestyle) |
| **BMI < 25 kg/m^2^ (n=5,294)** | | | | |
| Quartile 1 | 171 / 3,322 | Ref | Ref | Ref |
| Quartile 2 | 127 / 1,407 | 1.23 (0.84-1.80) | 1.17 (0.80-1.71) | 1.09 (0.75-1.58) |
| Quartile 3 | 41 / 417 | 0.95 (0.57-1.57) | 0.91 (0.56-1.49) | 0.85 (0.48-1.50) |
| Quartile 4 | 16 / 148 | 2.00 (0.90-4.44) | 1.75 (0.81-3.82) | 1.62 (0.67-3.83) |
| Continuous HR |  | 1.08 (0.97-1.20) | 1.06 (0.96-1.18) | 1.04 (0.92-1.18) |
| P Trend |  | 0.15 | 0.23 | 0.53 |
| **BMI ≥ 25 kg/m^2^ (n=8,334)** | | | | |
| Quartile 1 | 52 / 1,423 | Ref | Ref | Ref |
| Quartile 2 | 155 / 2,420 | 1.61 (1.07-2.43) | 1.60 (1.05-2.43) | 1.48 (0.98-2.23) |
| Quartile 3 | 225 / 2,491 | 2.21 (1.46-3.53) | 2.12 (1.39-3.25) | 2.00 (1.31-3.03) |
| Quartile 4 | 191 / 2,000 | 2.66 (1.60-4.45) | 2.61 (1.52-4.21) | 2.34 (1.42-3.86) |
| Continuous HR |  | 1.13 (1.07-1.21) | 1.12 (1.05-1.20) | 1.12 (1.05-1.19) |
| P Trend |  | <.0001 | 0.001 | 0.001 |
| Model Covariates: (1) fasting status (<6 hours/>=6 hours), age (continuous), sex (male/female), ethnicity(Non-Latino White/Non-Latino Black/Mexican American), (2) Model 1 + education (High school or less), health insurance coverage (yes/no), urbanization (% urban), (3) Model 2 + HEI scores (continuous), physical inactivity (% active yes/no), smoking status (current/former/never), alcoholic drinks per week (continuous), medication (any/none) | | | | |

| **Supplementary Table 3. Hazard Ratio and 95% CI of Cancer Mortality for Domain-Specific Variables of Multi-Systemic Biological Risk stratified by BMI (< 25 vs. ≥ 25 kg/m^2^), (n = 13,628), NHANES III, 1994-1998** | | | |
| --- | --- | --- | --- |
|  | HR (95%CI)  Model 1: Demographics | HR (95%CI)  Model 2: + SES | HR (95%CI)  Model 3: +Lifestyle |
| **BMI < 25 kg/m^2^ (Cancer deaths n=355/Total (n=5,294)** | | | |
| Immune Index | 1.25(1.02-1.52) | 1.22 (1.00-1.49) | 1.07 (0.88-1.30) |
| Metabolic Index | 1.01 (0.87-1.18) | 1.00 (0.86-1.17) | 1.02 (0.87-1.20) |
| Autonomic Index | 1.08 (0.85-1.38) | 1.05 (0.83-1.34) | 1.03 (0.81-1.31) |
| **BMI ≥ 25 kg/m^2^ (Cancer deaths n=623/Total n=8,334)** | | | |
| Immune Index | 1.34 (1.16-1.55) | 1.33 (1.14-1.54) | 1.26 (1.08-1.48) |
| Metabolic Index | 1.05 (0.96-1.14) | 1.04 (0.96-1.13) | 1.04 (0.96-1.14) |
| Autonomic Index | 1.26 (1.03-1.54) | 1.24 (1.01-1.52) | 1.23 (1.00-1.52) |
| Covariates: (1) fasting status(<6 hours/>=6 hours), age (continuous), sex (male/female), ethnicity (Non-Latino White/Non-Latino Black/Mexican American), (2) Model 1 + education (High school or less), health insurance coverage (Yes/No), urbanization (% urban), (3) Model 2 + HEI scores (continuous), physical inactivity (% active yes/no), smoking status (current/former/never), alcoholic drinks per week(continuous), medication (any/none). *This table includes the continuous hazard ratios for each domain, and all models include all three of the domain-specific index variables. | | | |

**Supplementary References**

1. Banga, S. & Chalfoun, N. Arrhythmias and Antiarrhythmic Drugs. in *Cardiology: An Integrated Approach* (ed. A, E.)

2. RF, L., DD, B., M, S. & JF., S. Vital Signs, Anthropometric Data, and Pain. in *DeGowin’s Diagnostic Examination,* 10e (eds. RF, L., DD, B., M, S. & JF., S.) (McGraw-Hill, 2015).

3. Chobanian, Aram V., George L. Bakris, Henry R. Black, William C. Cushman, Lee A. Green, Joseph L. Izzo Jr, Daniel W. Jones et al. "The seventh report of the joint national committee on prevention, detection, evaluation, and treatment of high blood pressure: the JNC 7 report." JAMA 289, no. 19 (2003): 2560-2571.

4. James, P. A. *et al.* 2014 Evidence-Based Guideline for the Management of High Blood Pressure in Adults. *JAMA* **311**, 507 (2014).

5. Stern, S. E. *et al.* Identification of individuals with insulin resistance using routine clinical measurements. *Diabetes* **54**, 333–9 (2005).

6. Ascaso, J. F. *et al.* Diagnosing insulin resistance by simple quantitative methods in subjects with normal glucose metabolism. *Diabetes Care* **26**, 3320–5 (2003).

7. Singh, B. & Saxena, A. Surrogate markers of insulin resistance: A review. *World J. Diabetes* **1**, 36–47 (2010).

8. Burtis, C. A. & Bruns, D. E. Reference Information. in *Tietz Fundamentals of Clinical Chemistry and Molecular Diagnostics* (Elsevier Health Sciences, 2014).

9. World Health Organization. Waist Circumference and Waist-Hip Ratio Report of a WHO Expert Consultation. *WHO Heal. Hum. Rights* (2011).

10. D, N., L.C., M. & S.J, M. Lab Tests. in *Guide to Diagnostic Tests* (eds. D, N., L.C., M. & S.J, M.) (McGraw-Hill, 2017).

11. Abramson, N. & Melton, B. Leukocytosis: basics of clinical assessment. *Am. Fam. Physician* **62**, 2053–60 (2000).

12. Pagana, K. D. & Pagana, T. J. *Mosby’s Manual of Diagnostic and Laboratory Tests-E-Book*. (Elsevier Health Sciences, 2017).

13. Greenland, P., Smith, S. C., Grundy, S. M. & Meisinger, C. Improving Coronary Heart Disease Risk Assessment in Asymptomatic People: Role of Traditional Risk Factors and Noninvasive Cardiovascular Tests. *Circulation* **104**, 1863–1867 (2001).
